# Machine Learning Approaches to Understanding and Predicting Patterns of Adherence

**DOI:** 10.1093/geroni/igab046.2117

**Published:** 2021-12-17

**Authors:** Shayok Chakraborty, Aditya Bhattacharya, Shubo Tian, Nelson Roque, Zhe He, Walter Boot

**Affiliations:** 1 Florida State University, Tallahassee, Florida, United States; 2 University of Central Florida, Orlando, Florida, United States

## Abstract

In cognitive training of older adults, adherence is a major challenge, but appropriate just-in-time adaptive interventions can improve adherence. To understand adherence patterns and predictors of adherence lapses, we aggregated data from two previous trials (N > 230) involving home-based cognitive interventions. This dataset, detailing 40,000 intervention interactions, contains information about intervention engagement and measures of objective and subjective cognitive performance, demographics, technology proficiency, and attitudes. Exploratory analyses were conducted to understand patterns and predictors of faltering adherence, using classification models, together with feature selection to remove redundant variables. Adherence behaviors in a week were predictive of quitting the following week. Game parameters such as the time of play were weak indicators of future playing patterns, whereas game success was a strong predictor of adherence. These and other useful observations will be incorporated in the design and development of the smart reminder system to be deployed in the APPT project.

